# Trends in enrollment, retention, and graduation of United States veterinary technicians/nurses schools

**DOI:** 10.3389/fvets.2024.1403799

**Published:** 2024-05-09

**Authors:** Lori R. Kogan, Wayne A. Jensen

**Affiliations:** College of Veterinary Medicine and Biomedical Sciences, Colorado State University, Fort Collins, CO, United States

**Keywords:** veterinary technician/nurse, salary, credentials, utilization, enrollment

## Abstract

**Objective:**

There is a significant shortage of veterinary technicians. To help address this issue, there has been a call to increase the capacity of United States VT/N educational programs. Yet, the current challenges within the field may be negatively impacting the number of people deciding to pursue VT/N certification. To assess this possibility, this study was designed to explore the enrollment, retention, and graduation trends within United States VT/N educational programs. Explore the trends between 2018 and 2022 in enrollment, retention, and graduation of veterinary technicians/nurses (VT/N) at educational programs located in the United States.

**Sample:**

Educators and administrators working in United States VT/N educational programs.

**Procedures:**

An electronic survey distributed via an anonymous link within emails sent from the Association of Veterinary Technician Educators.

**Results:**

A total of 82 responses from educators and administrators working in United States VT/N educational programs were received. Forty-three percent of participants indicated a decrease in student enrollment in the last 5 years. The factors seen to have the largest significant impact were “More potential students not convinced being credentialed will lead to a difference in job duties when compared to non-credentialed work,” “More potential students who do not think being credentialed will lead to a substantial increase in pay when compared to non-credentialed work” and “More potential students not willing/able to invest the time needed to become credentialed.” A total of 60% reported an increase in retention efforts within the last 5 years. The services most commonly reported as increasing included mental health support and academic mentoring.

**Conclusions and clinical relevance:**

This study suggests that an increased number of potential VT/N students are deciding that being credentialed is not worth the time or money. While additional resources directed toward recruitment and retention are needed within VT/N educational programs, without systematic changes within the field, it is likely that there will be a continued decline in the number of interested applicants.

## 1 Introduction

Veterinary technicians/nurses (VT/N) are instrumental in the provision of patient care, client education and practice efficiency (1). It has been estimated that one additional VT/N is associated with a 20.5% increase in hospital revenue (2). The current ratio of VT/Ns to veterinarians for companion animal practices is about 2:1, yet to maximize productivity and revenue, it has been suggested this ratio should be at least 4:1 (3). In fact, a recent study found the technician-to-veterinarian labor ratio (TVLR) of 9:1 is best for revenue while a TVLR of 10:1 is optimal for productivity (2).

These figures suggest a significant shortage of VT/Ns. In 2019, there were an estimated 118,000 VT/Ns in the US. What is unknown is how many of these individuals are credentialed, but an estimate of credentialed veterinary technicians in 2017 was only around 70,000 (4). There are far fewer veterinary technician specialists, with estimates of only 1,100 in 2017 (4). It is clear that there is a need for additional VT/Ns; it has been estimated that 133,000 credentialed companion animal VT/Ns will be needed by 2030 to meet the growing demand (5).

Given these figures, it is not surprising that employment opportunities for VT/Ns are expected to grow 21% from 2022 to 2032, much faster than most other occupations, with approximately 14,800 openings for VT/Ns projected annually over the next decade (6). To help meet this demand, there are approximately 230 AVMA accredited Veterinary Technology Programs in the US. (7). Credentialed VT/Ns must graduate from programs accredited by College of Veterinary Technician Education Association (CVTEA). Graduates of these programs are eligible to take the Veterinary Technician National Exam (VTNE). Nearly all US states require a person to be a graduate of a CVTEA-accredited veterinary technology program to be eligible for credentialing in their state (8). While many uncredentialed technicians are quite capable and skilled, a formal education program helps establish a consistent predictable baseline of knowledge (3). Knowing what is included in the curricula of accredited VT/N programs can help veterinarians better utilize their VT/N’s skills and expertise.

While most VT/Ns programs are traditional in-person, there are a number of AVMA accredited distance learning VT/N programs (9). Most VT/N schools are public community colleges (52%), followed by for-profit private institutions (24%), public universities (15%) and not-for-profit private universities (10%) (7). The majority of these schools offer an associate degree (94%), most often in applied science (74%) or science (21%). The cost (tuition, fees, books) of these programs ranges from an average of $17,335 for in-state to $28,935 for out-of-state. Fifteen percent of these programs offer a bachelor’s degree (some in addition to an associate’s degree), with most of these schools (76%) offering a bachelor’s in science. The average cost (tuition, fees, books) of these bachelor’s programs range from $32,122 for in-state to $58,336 for out-of-state (7).

In the academic year 2020–2021, AVMA accredited Veterinary Technology Programs in the US graduated approximately 4,813 students (52 more students than 2019–2020) with an associate degree and 471 students with a bachelor’s degree (5 more students than 2019–2020) (7). Over the last 6 years, the number of students sitting for the VTNE has been around 7,000, far short of the number needed to fill current estimated needs (3).

To address this shortage, it has been suggested that there should be an increase in the capacity of US VT/N educational programs by increasing the number of VT/N programs and/or increasing enrollment within the existing programs (3). It is possible however, that despite the shortage and the projected positive job market, the current challenges within the field are impacting the number of people deciding to pursue VT/N certification. To explore this possibility, we designed a study to explore the enrollment, retention, and graduation trends within US VT/N educational programs.

## 2 Materials and methods

We created an online survey and partnered with the Association of Veterinary Technician Educators to distribute the survey to VT/N school. Participants could anonymously access the survey from June 15, 2023 through August 30, 2023. The study was categorized as exempt by Colorado State University’s Institutional Review Board. The survey asked participants to report changes over the last 5 years in the following areas (See Appendix for full survey):

- Student enrollment
- Amount of money/time allocated toward recruitment
- Percent of filled seats
- Graduation rate
- Student retention efforts
- Student services
- Veterinary Technician National Examination (VTNE) pass rate for first time test takers

Descriptive statistics and chi-square tests were performed with SPSS, version 28. Chi square was used to assess differences between years for percent of seats filled and NVTE pass rate, with significance level set at 0.05.

## 3 Results

We received a total of 82 responses, 36 (44%) from program directors; 35 (43%) from faculty, 4 (5%) from program administrators, and 7 (9%) from ‘other’ (e.g., managers, developers, coordinators).

### 3.1 Student enrollment

Respondents were asked to report how student enrollment had changed in the last 5 years using a 7-point Likert scale from 1 = decreased significantly to 7 = increased significantly (*n* = 82). We found that 43% reported a decrease in student enrollment (Table 1).

**Table 1:** Change in student enrollment in the last 5 years.

	*N*	%
Decreased significantly	5	6.1
Decreased moderately	13	15.9
Decreased slightly	17	20.7
Stayed the same	16	19.5
Increased slightly	15	18.3
Increased moderately	9	11.0
Increased significantly	7	8.5
Total	82	100.0

### 3.2 Percent of filled seats and reasons for a decrease in enrollment

**Figure 1:**
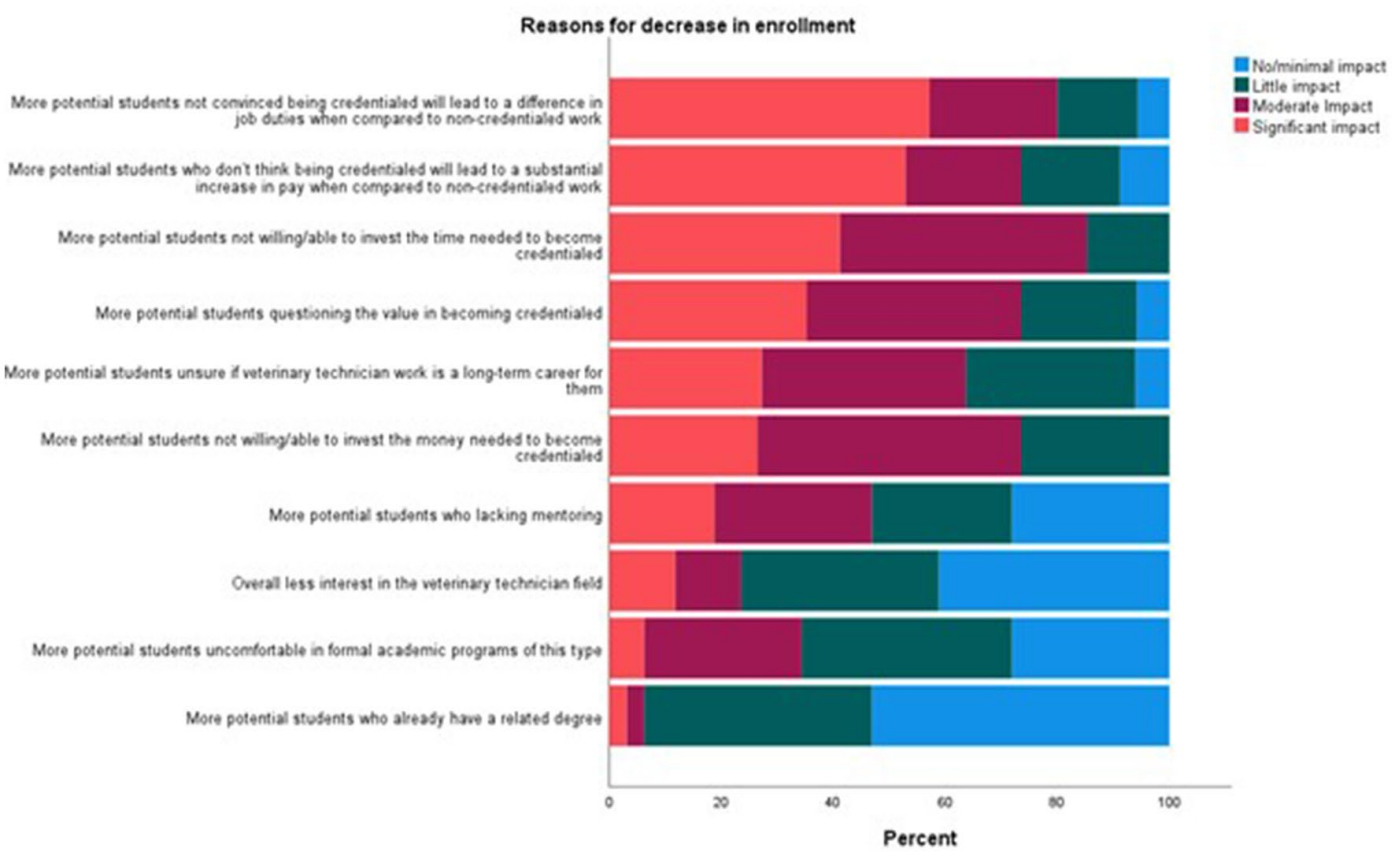
Percent of filled seats within VT/N programs over the last five years.

Participants were asked to indicate the percentage of available seats they filled each year between 2018 and 2022 (*n* = 81). The percentage of respondents who reported 90–100% of their seats were filled for the years 2022, 2021, and 2020 remained consistent (56–57%), but were lower than 2019 (73%, *X*^2^ < 0.001) or 2018 (74%, *X*^2^ < 0.001) (Figure 1).

**Figure 2:**
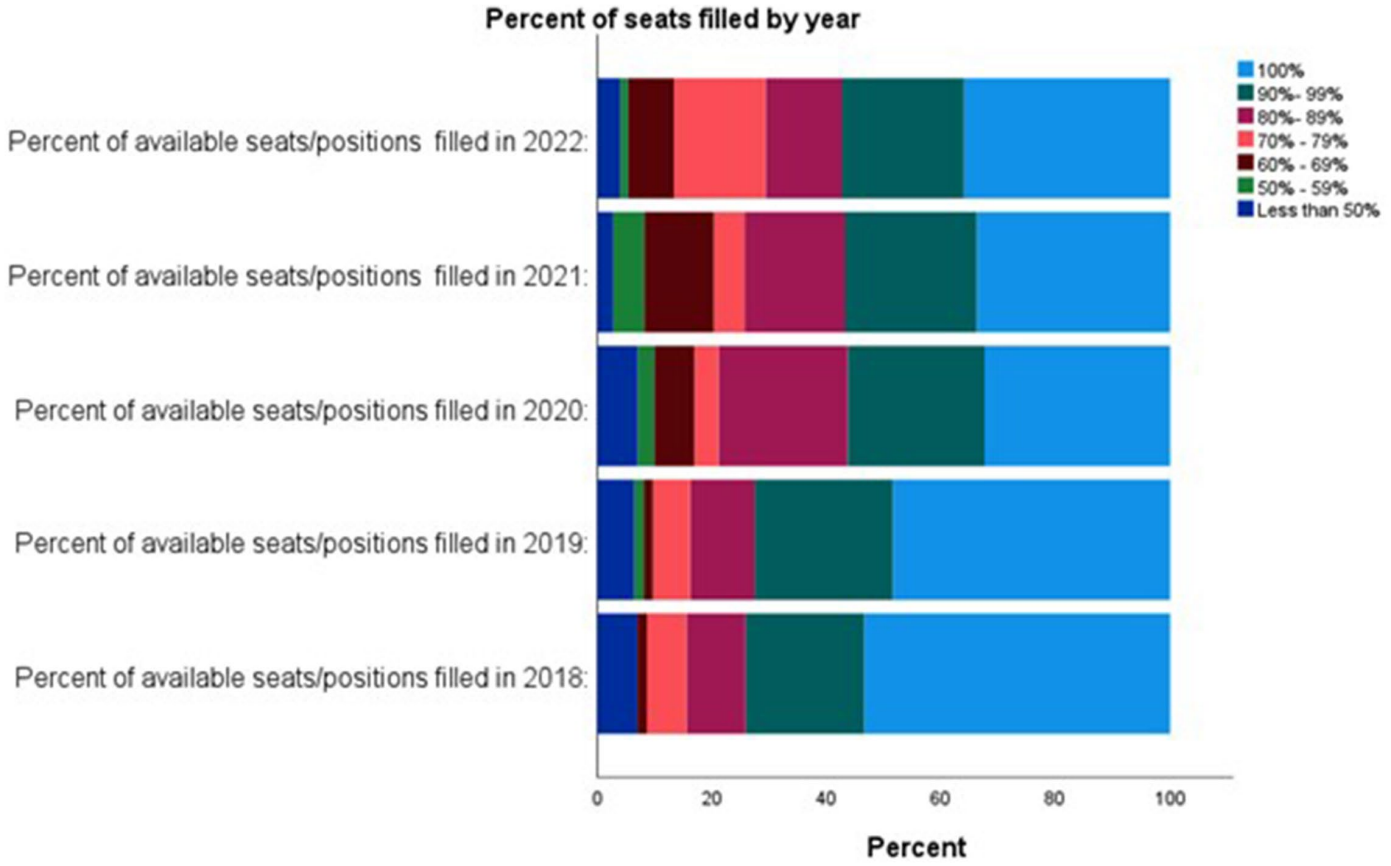
Perceived reasons for a decrease in enrollment in United States VT/N programs.

The participants who reported a decrease in enrollment were asked to indicate the perceived impact of several factors (*n* = 35) using a 4-point Likert scale with 1 = no/minimal impact and 4 = significant impact. The factors that had the largest significant impact were “More potential students not convinced being credentialed will lead to a difference in job duties when compared to non-credentialed work” (significant impact: 57%), “More potential students who do not think being credentialed will lead to a substantial increase in pay when compared to non-credentialed work” (significant impact: 53%), and “More potential students not willing/able to invest the time needed to become credentialed” (significant impact: 41%) (Figure 2).

### 3.3 Amount of money/time allocated toward recruitment

A 7-point Likert scale from 1 = decreased significantly to 7 = increased significantly was used when participants were asked about any change in the amount of money/time their school has allocated toward recruitment in the last 5 years (*n* = 67). A total of 43% of participants reported an increase in money/time allocated toward recruitment (Table 2).

**Table 2:** Change in amount of time and/or money school has allocated toward recruitment effort in the last 5 years.

	*N*	%
Yes, decreased significantly	3	4.5
Yes, decreased slightly	6	9.0
No, stayed the same	29	43.3
Yes, increased slightly	17	25.4
Yes, increased moderately	8	11.9
Yes, increased significantly	4	6.0
Total	67	100.0

### 3.4 Graduation rate

**Figure 3:**
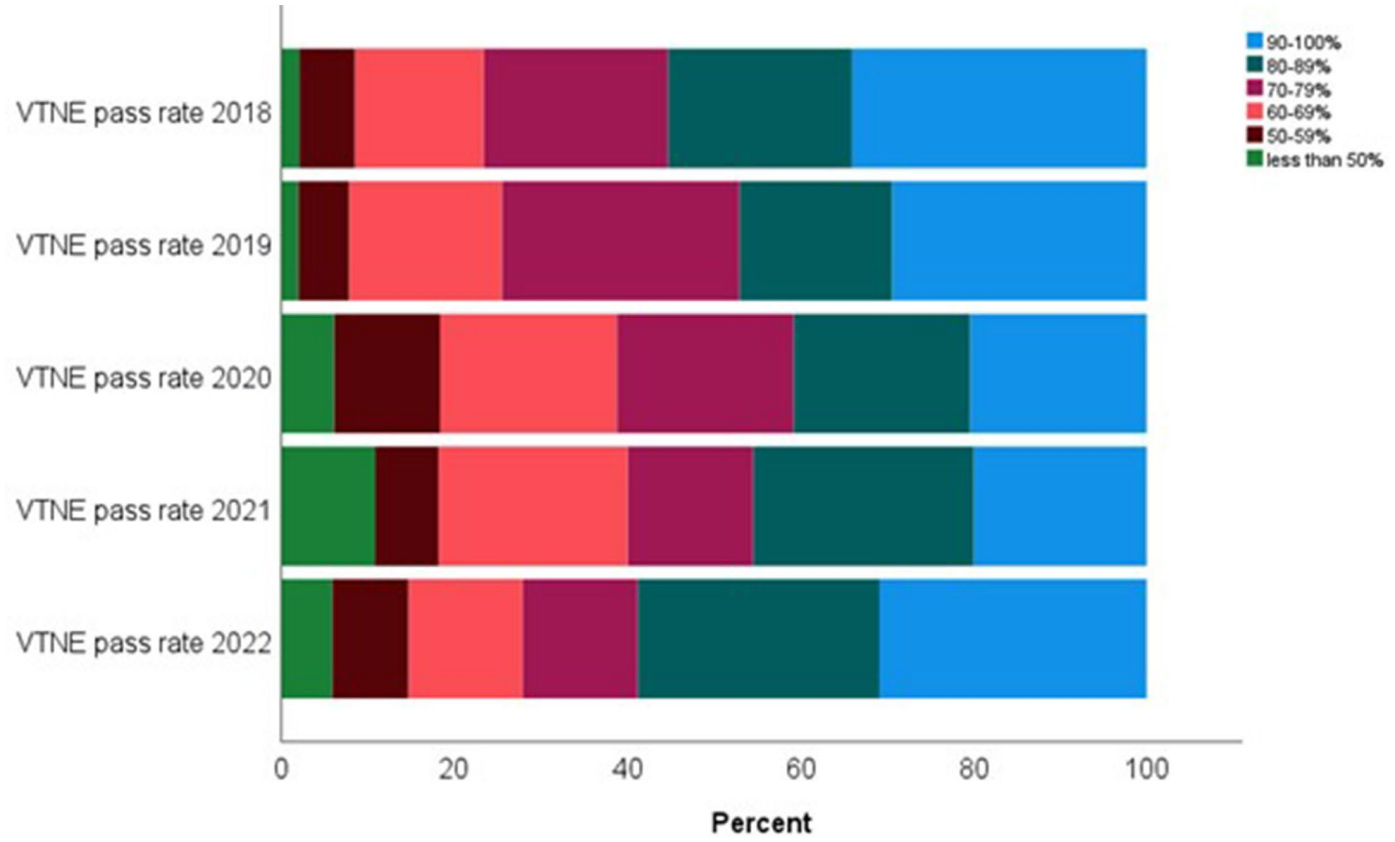
Reasons for a decrease in graduation rate within United States VT/N programs.

When participants were asked about any changes in graduation rates over the last 5 years (*n* = 78) using a 7-point Likert scale with 1 = decreased significantly and 7 = increased significantly, 39% noted no difference, while 38% noted a decrease and 23% reported an increase (Table 3). Participants who reported a decrease in graduation were asked to indicate the perceived impact of several factors (*n* = 30) using a 4-point Likert scale with 1 = no/minimal impact and 4 = significant impact. The factors seen as having the largest significant impact included “More students with mental health challenges” (significant impact, 64%), “More students unable to pass their classes” (significant impact, 48%), and “More students not willing/able to invest the time needed” (significant impact, 31%) (Figure 3).

**Table 3:** Change in graduation rate over the last 5 years.

	*N*	%
Yes, decreased significantly	4	5.1
Yes, decreased moderately	10	12.8
Yes, decreased slightly	16	20.5
No, stayed the same	30	38.5
Yes, increased slightly	10	12.8
Yes, increased moderately	5	6.4
Yes, increased significantly	3	3.8
Total	78	100.0

### 3.5 Retention efforts

**Figure 4:**
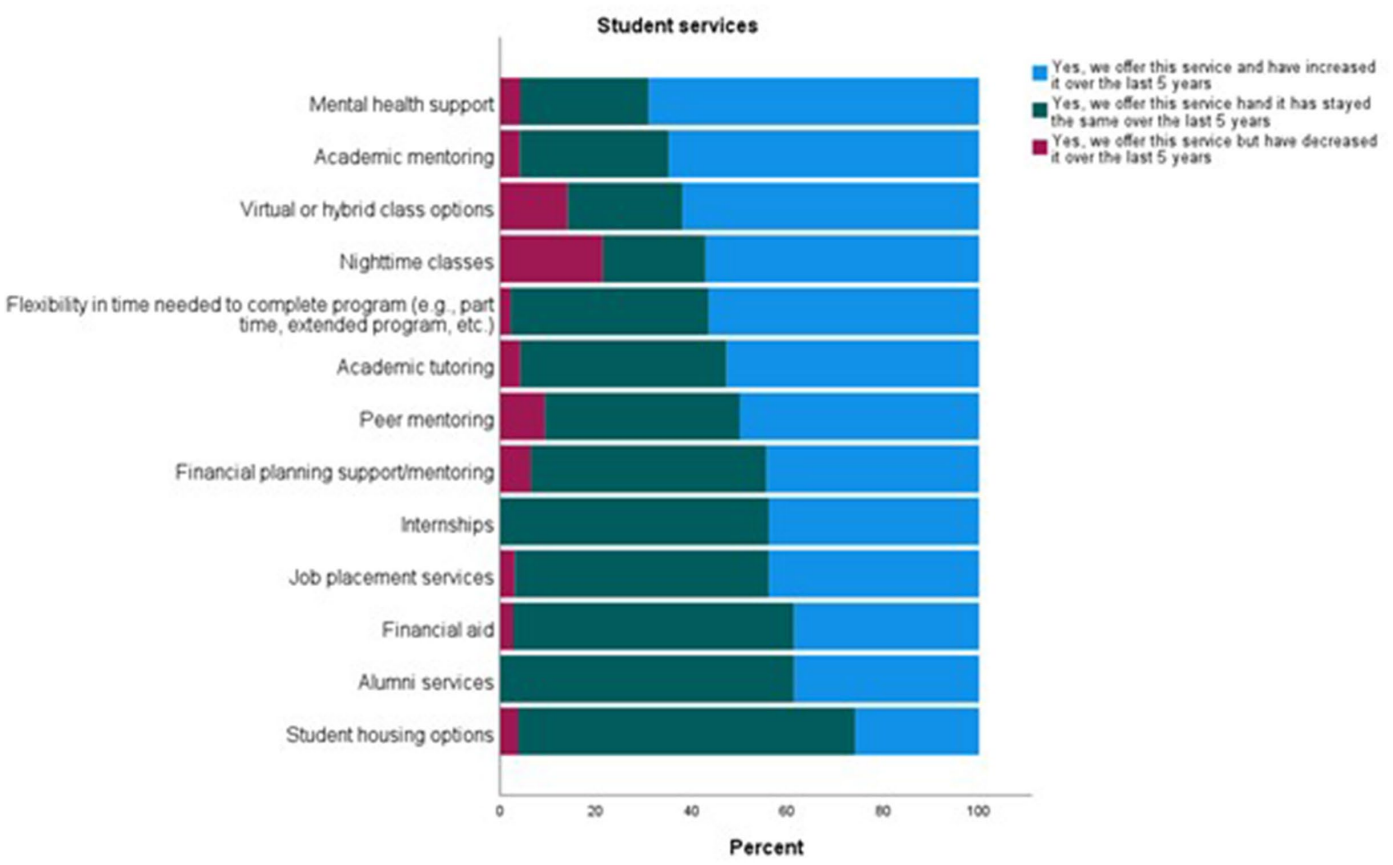
Student services within United States VT/N programs.

Participants were asked to describe how their student retention efforts had changed over the last 5 years (*n* = 76) using a 7-point Likert scale with 1 = decreased significantly and 7 = increased significantly. A total of 60% reported an increase in retention efforts (Table 4). To obtain more details about student services offerings, participants were asked to indicate which services they provide, and for those services they provide, to indicate if the service has increased, decreased, or remained the same over the last 5 years. The participants reported offering all the services listed. The services most commonly reported as increasing within the last 5 years include “mental health support” (69%), “academic mentoring” (65%), and “virtual or hybrid class options” (62%) (Figure 4).

**Table 4:** Change in student retention efforts in the last 5 years.

	*N*	%
Yes, decreased moderately	4	5.3
Yes, decreased slightly	5	6.6
No, stayed the same	21	27.6
Yes, increased slightly	20	26.3
Yes, increased moderately	16	21.1
Yes, increased significantly	10	13.2
Total	76	100

**Figure 5:**
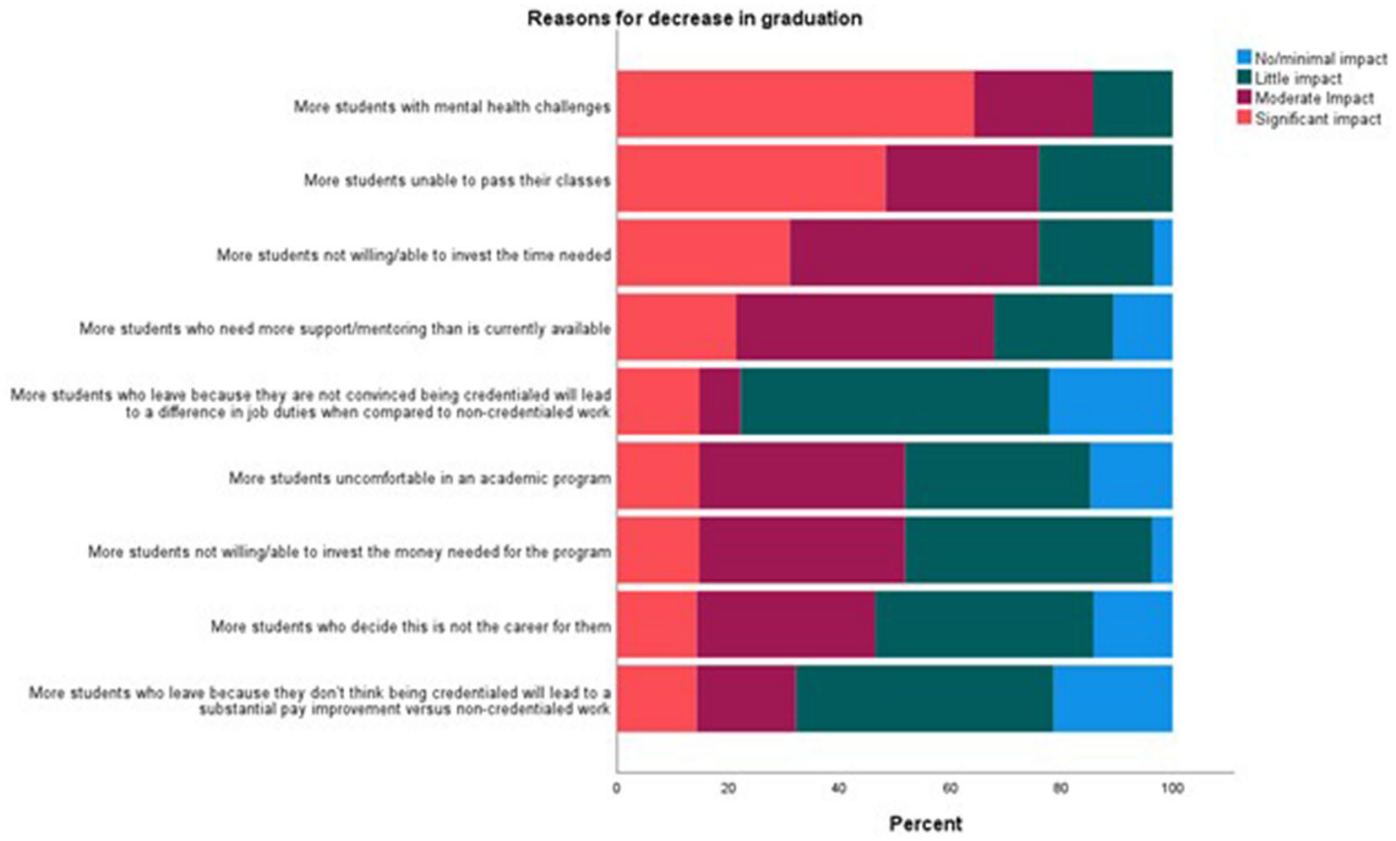
Veterinary Technician National Examination (VTNE) pass rate of VT/N programs within the United States.

Lastly, we asked participants about their school’s VTNE pass rate for first time test takers over the last 5 years. A pass rate between 90 and 100% was reported by approximately 30% of respondents for the years 2022 (21/68, 31%), 2019 (15/51, 29%), and 2018 (16/47, 34%). A pass rate between 90–100% was reported by 20% of respondents for years 2021 (11/55) which was significantly different than 2018 (*p* = 0.019) and 20% for 2020 (10/49), also significantly different than 2018 (*p* = 0.007) (Figure 5).

## 4 Discussion

This study provides a snapshot of the enrollment, recruitment, and retention trends within US veterinary technician schools. We found that 43% of participants reported a decrease in student enrollment. This is in spite of the fact that the same percentage (43%) reported an increase in money and time allocated to recruitment efforts. As a result, schools reported fewer seats filled in the last 3 years when compared to earlier years. Although the actual reasons for this decline are unknown, participants speculated that it is largely due to potential students being unconvinced that credentialing will lead to a substantial increase in salary or a difference in job duties and responsibilities. As a result, it would appear that more potential students are deciding that being credentialed is not worth the time or money.

The concern about salary has been echoed among veterinary technicians working in the field. Both the 2016 and 2022 NAVTA surveys, for example, reported that low salary is seen as the most challenging aspect of technicians’ jobs (10, 11). Although the 2022 NAVTA survey found credentialed veterinary technicians earn an average of $8.20 more per hour than non-credentialed ($26.80 versus $18.60), many technicians do not perceive a noticeable difference. For example, only 9% of respondents to the 2022 NAVTA survey reported feeling that veterinary technician/nurses with a bachelor’s degree earn more than veterinary technician/nurses with an associate’s degree. Exacerbating the issue of low wages, over a third of veterinary technicians have student loan debt, with an average amount of $29,700 (11).

In addition to concerns about low salary, many potential students appear unconvinced that becoming credentialed will lead to a change in job duties and responsibilities. Again, these concerns appear legitimate. Over half (54%) of those surveyed in the 2022 NAVTA study indicated feeling that credentialed veterinary technician/nurses and uncredentialed support staff are trained to perform the same job duties. Furthermore, 1 in 5 reported feeling they are not utilized to their fullest potential, with primary barriers including a lack of trust by veterinarians in veterinary technician/nurses’ skills, an unwillingness of veterinarians to let veterinary technician/nurses complete tasks they are trained to perform, and uncredentialed technicians permitted to do veterinary technician/nurses tasks. The long-standing practice of “title creep,” where veterinary assistants are misidentified as veterinary technicians and trained to do the work of a credentialed VT/N, may contribute to VT/N disengagement and decreased interest in obtaining additional training or certification. Despite the fact that these barriers could be addressed with training and education, 20% of technicians reported feeling that nothing is being done at their hospitals in an effort to improve their utilization (11). Clarifying nomenclature by educating all hospital staff on the different skills and knowledge credentialed VT/Ns have compared to veterinary assistants and VTS to VT/N, as well as creating a system to easily identify everyone’s role, could help veterinarians best utilize each staff member. Without these changes, factors related to wages and utilization make it difficult for prospective technician students to feel the money and time invested in becoming credentialed will actually pay off in terms of salary and increased responsibilities.

Our study also assessed retention efforts and found that a majority of respondents reported an increase in retention efforts. The most common services reported to have increased within the last 5 years include mental health support (69%), academic mentoring (65%), and virtual or hybrid class options (62%).

The prevalence of mental health challenges among college student populations has nearly doubled over the last 10 years, making it a top concern in higher education (12, 13). During the 2020–2021 school year, more than 60% of college students met the criteria for at least one mental health problem, a nearly 50% increase from 2013 (14). The mental health of college students has worsened among all racial/ethnic groups, with a 135% increase in depression and 110% increase in anxiety from 2013 to 2021 (14).

One potential partial explanation for this increase is a change in students’ openness to report symptoms and willingness to seek help. Lipson et al. found that 37% of college students reported receiving mental health counseling in 2021, an increase of 7% from 2020 (14). This increase in the demand for mental health services exceeds many schools’ available resources (15). It would appear that veterinary technician schools are experiencing a similar increase in demand for mental health services. As the need for these services continues to grow, it is suggested that telehealth mental health services might be an option. Telehealth offers several potential advantages over in-person treatment for college students including increased ability to access care and the option for students to attend sessions in the privacy of their own homes (16, 17).

The second most common student service reported to have increased over the last 5 years is academic mentoring. Perhaps this is unsurprising given that the second most common reason given for decreased graduation rate is an increase in the number of students unable to pass their classes. There are likely many reasons for this trend, including the changing demographics of college students.

The college population has become increasingly diverse, with growing numbers of “nontraditional” students; 40% of current college students are now over the age of 25, 44% are students of color, and 34% are first-generation students (18). This translates into more college students juggling work, familial, and other responsibilities with academics (19). In an effort to better support students, 65% of participants noted an increase in academic mentoring. In addition to academic mentoring, veterinary technician schools may benefit from a coaching program. While only recently utilized within medical education, coaching, helping individuals achieve their personal best, has been used successfully for decades within sports and business programs (20). Professional coaching differs from traditional tutoring or academic mentoring in that it does not focus on knowledge transmission, advice, or counseling. Instead, coaching focuses on individualized feedback of observed behavior and the use of stimulating and challenging observations and interactions to help students achieve their full potential (21). Perhaps it is possible to enlist the help of coaches to help veterinary technician students excel, not only at technical skills, but ways to negotiate for higher salaries and additional responsibilities.

While supportive student services are needed within veterinary technician schools to help enrolled students excel, without systematic changes within the field, it is likely that there will be a continued decline in the number of interested applicants. Within the field, several options to increase veterinary technician salary and utilization are being discussed. Many states have enacted title protection, for example, that restricts use of the term “veterinarian technician” to credentialed VT/Ns, a change that was supported by 90 and 83% of respondents to the 2016 and 2022 NAVTA surveys, respectively (10, 11). Restricting performance of certain tasks to credentialed VT/Ns was also supported by approximately 65% of respondents to the 2016 NAVTA survey (10). This question was not asked in the 2022 NAVTA survey although respondents identified the use of uncredentialed staff to do VT/N tasks and one of the top barriers to better utilization (11). Unfortunately, on-the-job experience does not appear to have a significant impact on credentialed VT/N salaries as evidenced by the fact that those with 6–10 years of experience receive only $1.40 more per hour than those with less than 1 year of experience (11).

The lack of a clear path for credentialed VT/Ns that leads to greater salary and responsibility is likely to negatively impact both recruitment and retention. Current and future career options for VT/Ns to help address this issue include advanced training to become a Veterinary Technician Specialists (VTS) or a Veterinary Professional Associate (VPA). The proposed VPA position is a midlevel practitioner role, similar to a physician assistant in human medicine (22). Currently, establishing a VPA position is being considered in both Florida and Colorado (22). When asked their views of VPA type position, 79% of VT/Ns reported being interested (4).

It should be noted there are several limitations to this study. First, the data analyzed in this study span time including the COVID-19 pandemic. In addition, because the survey was anonymous, it is not possible to determine if more than one person per school completed the survey. In the future, this might be prevented by targeting specific individuals within each institution. Our sample consisted of a small percentage of veterinary technician schools, so caution is suggested when generalizing to other schools since we cannot be certain that our sample represents the overall veterinary technician school population. Potential response bias is another limitation. It is possible that individuals who feel strongly about the topic, either positive or negative, might have been more likely to respond to the survey. This study, however, offers useful insights into the current trends of veterinary technician schools. In summary, regardless of the specific path, it is clear that the field of veterinary technicians/nurses is at a tipping point and will not be able to maintain the status quo. This study highlights the fact that it is time for a change.

## Data availability statement

The raw data supporting the conclusions of this article will be made available by the authors, without undue reservation.

## Ethics statement

The studies involving humans were approved by Colorado State University Institutional Review Board. The studies were conducted in accordance with the local legislation and institutional requirements. The ethics committee/institutional review board waived the requirement of written informed consent for participation from the participants or the participants’ legal guardians/next of kin because survey was anonymous, approved by IRB.

## Author contributions

LK: Conceptualization, Data curation, Formal analysis, Investigation, Methodology, Writing – original draft, Writing – review & editing. WJ: Conceptualization, Writing – review & editing.
