# Supplementary material for: Trends in enrollment, retention, and graduation of United States veterinary technicians/nurses schools
Source: Front Vet Sci. 2024 May 9;11:1403799. doi: 10.3389/fvets.2024.1403799 (PMC11111897; doi:10.3389/fvets.2024.1403799)
Supplement: Supplementary file 1 [file Data_Sheet_1.docx]

Veterinary Technician Programs

Dear Participant,


 My name is Lori Kogan and I am a researcher from Colorado State University in the Clinical Sciences department. The title of our project is Veterinary Technician Programs - recruitment and retention. In an effort to gather data related to the veterinary technician shortage, we are reaching out to all veterinary technician programs to better understand recent enrollment and retention trends. To this end, we ask you to complete the following brief (less than 10 minutes) anonymous survey. Results from this survey will be aggregated and a final report will be shared with all veterinary technician programs.


Upon completion of the survey, you will be directed to a new window where you can enter your name into a raffle for a $100 Amazon gift card. In this way, your survey remains totally anonymous.

 
 
Fine print: Your participation in this research is voluntary. If you decide to participate in the study, you may stop the survey and close the website at any time, without penalty. We will not collect your name or personal identifiers. When we report and share the data to others, we will combine the data from all participants. Results of this survey will be used for research purposes. Information from this survey could be used for future research studies or distributed to another investigator for future research studies without additional informed consent from you or the legally authorized representative. While there are no direct benefits to you, the knowledge we gain will help us with our efforts to better support the field of veterinary technicians.

 It is not possible to identify all potential risks in research procedures, but the researcher(s) have taken reasonable safeguards to minimize any known and potential (but unknown) risks. If you have any questions about the research, please contact Lori Kogan at lori.kogan@colostate.edu. If you have any questions about your rights as a volunteer in this research, contact the CSU IRB at: CSU_IRB@colostate.edu; 970-491-1553. If you consent to complete this survey, please click “Yes I consent” below, to begin the survey.

- Yes, I consent to participating in this survey
- No, I do not consent to participating in this survey

What is your role within the VT program?

- Program Director
- Program Administrator
- Other (please explain): __________________________________________________

Please answer the following questions about enrollment

Please indicate if/how your student enrollment has changed in the last 5 years:

- Decreased significantly
- Decreased moderately
- Decreased slightly
- Stayed the same
- Increased slightly
- Increased moderately
- Increased significantly

Please indicate the percent of available seats/positions you filled for the following years.

Percent of available seats/positions you filled in 2022:

- 100%
- 90%- 99%
- 80%- 89%
- 70% - 79%
- 60% - 69%
- 50% - 59%
- Less than 50%
- Don’t know

Percent of available seats/positions you filled in 2021:

- 100%
- 90% - 99%
- 80% - 89%
- 70% - 79%
- 60% - 69%
- 50% - 59%
- Less than 50%
- Don’t know

Percent of available seats/positions you filled in 2020:

- 100%
- 90% - 99%
- 80% - 89%
- 70% - 79%
- 60% - 69%
- 50% - 59%
- Less than 50%
- Don’t know

Percent of available seats/positions you filled in 2019:

- 100%
- 90% - 99%
- 80% - 89%
- 70% - 79%
- 60% - 69%
- 50% - 59%
- Less than 50%
- Don’t know

Percent of available seats/positions you filled in 2018:

- 100%
- 90% - 99%
- 80% - 89%
- 70% - 79%
- 60% - 69%
- 50% - 59%
- Less than 50%
- Don’t know

We are hoping to understand the reasons for a decline in enrollment. Please indicate to what degree you feel the following aspects are negatively impacting enrollment:

|  | No/minimal impact | Little impact | Moderate Impact | Significant impact | Don't know |
| --- | --- | --- | --- | --- | --- |
| More potential students not willing/able to invest the money needed to become credentialed |  |  |  |  |  |
| More potential students not willing/able to invest the time needed to become credentialed |  |  |  |  |  |
| More potential students uncomfortable in formal academic programs of this type |  |  |  |  |  |
| More potential students who don’t think being credentialed will lead to a substantial increase in pay when compared to non-credentialed work |  |  |  |  |  |
| More potential students not convinced being credentialed will lead to a difference in job duties when compared to non-credentialed work |  |  |  |  |  |
| More potential students unsure if veterinary technician work is a long-term career for them |  |  |  |  |  |
| More potential students questioning the value in becoming credentialed |  |  |  |  |  |
| Overall less interest in the veterinary technician field |  |  |  |  |  |
| More potential students who already have a related degree |  |  |  |  |  |
| More potential students who lacking mentoring |  |  |  |  |  |

Other factors you feel are negatively impacting enrollment:

________________________________________________________________

Comments about any changes/trends you have witnessed in student enrollment:

________________________________________________________________

 Please answer the following questions about recruitment

Has the amount of time and/or money your school has allocated towards recruitment effort changed in the last 5 years?

- Yes, decreased significantly
- Yes, decreased moderately
- Yes, decreased slightly
- No, stayed the same
- Yes, increased slightly
- Yes, increased moderately
- Yes, increased significantly
- Don’t know

Comments about changes to your student recruitment efforts:

________________________________________________________________

Please answer the following questions about graduation

Has your graduation rate changed over the last 5 years?

- Yes, decreased significantly
- Yes, decreased moderately
- Yes, decreased slightly
- No, stayed the same
- Yes, increased slightly
- Yes, increased moderately
- Yes, increased significantly
- Don’t know

We are hoping to understand the reasons for a decline in graduation rates. Please indicate to what degree you feel the following aspects negatively impact graduation:

|  | No/minimal impact | Little impact | Moderate Impact | Significant impact | Don't know |
| --- | --- | --- | --- | --- | --- |
| More students not willing/able to invest the money needed for the program |  |  |  |  |  |
| More students not willing/able to invest the time needed |  |  |  |  |  |
| More students uncomfortable in an academic program |  |  |  |  |  |
| More students unable to pass their classes |  |  |  |  |  |
| More students with mental health challenges |  |  |  |  |  |
| More students who leave because they don’t think being credentialed will lead to a substantial pay improvement versus non-credentialed work |  |  |  |  |  |
| More students who leave because they are not convinced being credentialed will lead to a difference in job duties when compared to non-credentialed work |  |  |  |  |  |
| More students who decide this is not the career for them |  |  |  |  |  |
| More potential students who leave because they question the value in becoming credentialed |  |  |  |  |  |
| More students who need more support/mentoring than is currently available |  |  |  |  |  |

Other factors you feel are negatively impacting graduation rates:

________________________________________________________________

Comments about any changes/trends you have witnessed in student graduation:

________________________________________________________________

Please answer the following questions about retention

Have your student retention efforts changed in the last 5 years?

- Yes, decreased significantly
- Yes, decreased moderately
- Yes, decreased slightly
- No, stayed the same
- Yes, increased slightly
- Yes, increased moderately
- Yes, increased significantly
- Don’t know

Comments about any changes/trends you have witnessed in your student retention efforts:

________________________________________________________________

Please indicate which of the following student services your school offers and how they have changed over the last 5 years.

|  | Yes, we offer this service and have increased it over the last 5 years | Yes, we offer this service hand it has stayed the same over the last 5 years | Yes, we offer this service but have decreased it over the last 5 years | No, but we are planning to offer | No, and we have no plans to offer | Don't know |
| --- | --- | --- | --- | --- | --- | --- |
| Academic mentoring |  |  |  |  |  |  |
| Financial planning support/mentoring |  |  |  |  |  |  |
| Financial aid |  |  |  |  |  |  |
| Mental health support |  |  |  |  |  |  |
| Flexibility in time needed to complete program (e.g., part time, extended program, etc.) |  |  |  |  |  |  |
| Nighttime classes |  |  |  |  |  |  |
| Virtual or hybrid class options |  |  |  |  |  |  |
| Peer mentoring |  |  |  |  |  |  |
| Academic tutoring |  |  |  |  |  |  |
| Job placement services |  |  |  |  |  |  |
| Alumni services |  |  |  |  |  |  |
| Internships |  |  |  |  |  |  |
| Student housing options |  |  |  |  |  |  |

Other student services your school offers:

________________________________________________________________

Comments about any changes/trends you have witnessed in student services:

________________________________________________________________

Please indicate the percent of students (first time test takers) who have passed the VTNE for the following years.

Percent of students (first time test takers) who have passed the VTNE in 2022:

- 100%
- 90% - 99%
- 80% - 89%
- 70% - 79%
- 60% - 69%
- 50% - 59%
- Less than 50%
- Don’t know

Percent of students (first time test takers) who have passed the VTNE in 2021:

- 100%
- 90% - 99%
- 80% - 89%
- 70% - 79%
- 60% - 69%
- 50% - 59%
- Less than 50%
- Don’t know

Percent of students (first time test takers) who have passed the VTNE in 2020:

- 100%
- 90% - 99%
- 80% - 89%
- 70% - 79%
- 60% - 69%
- 50% - 59%
- Less than 50%
- Don’t know

Percent of students (first time test takers) who have passed the VTNE in 2019:

- 100%
- 90% 99%
- 80% - 89%
- 70% - 79%
- 60% - 69%
- 50% - 59%
- Less than 50%
- Don’t know

Percent of students (first time test takers) who have passed the VTNE in 2018:

- 100%
- 90% - 99%
- 80% - 89%
- 70%-79%
- 60% - 69%
- 50% - 59%
- Less than 50%
- Don’t know

Comments about any changes/trends you have witnessed in VTNE pass rates:

________________________________________________________________

Additional comments about student trends:

________________________________________________________________

Thank you for your time!
